# Evaluation of Physical Properties of Zirconia Suspension with Added Silane Coupling Agent for Additive Manufacturing Processes

**DOI:** 10.3390/ma15041337

**Published:** 2022-02-11

**Authors:** Jae-Gon Jang, Jin-Ho Kang, Kwang-Bum Joe, Kumaresan Sakthiabirami, Kyoung-Jun Jang, Mee-Jin Jun, Gye-Jeong Oh, Chan Park, Sang-Won Park

**Affiliations:** 1 Department of Prosthodontics, School of Dentistry, Chonnam National University, Gwangju 61186, Korea; jangjaegon@naver.com (J.-G.J.); jhk.bme1002@gmail.com (J.-H.K.); sakthikarthi.dentist@gmail.com (K.S.); zircong@naver.com (K.-J.J.); upgradepc@hanmail.net (C.P.); 2 School of Dentistry, Chonnam National University, Gwangju 61186, Korea; poopoopooy@naver.com; 3 Biomedical Evaluation and Research Centre, School of Dentistry, Chonnam National University, Gwangju 61186, Korea; timer22@naver.com; 4 Department of Dental Hygiene, Gwangju Health University, Gwangju 62287, Korea; jmejin@hanmail.net

**Keywords:** additive manufacturing, digital light processing (DLP) technology, zirconia suspension, silane coupling agent, physical property

## Abstract

In this study, we have analysed the effects of a silane coupling agent on the volume fraction of zirconia for digital light processing (DLP)-based additive manufacturing processes. Zirconia suspension was prepared by the incorporation of silane-modified zirconia particles (experimental group) or untreated zirconia particles (control group). Furthermore, the control and experimental group were subdivided into three groups based on the volume fraction (52, 54, and 56 vol%) of zirconia particles. The disk-shaped zirconia samples were 3D (three-dimensional) printed using the DLP technique and their physical and mechanical properties were evaluated. The addition of a silane coupling agent to the zirconia samples was found to have influence of about 6% on the hardness and biaxial flexural strength. Moreover, the decrease in minute air gaps inside the zirconia layers significantly increased the material density (visualized from the microstructure analysis). Thus, from this study, it was established that the silane-modified zirconia particles had a positive effect on the physical properties of the zirconia parts.

## 1. Introduction

Various materials have been used for prostheses in dental clinics, such as titanium, cobalt–chromium, zirconia, and glass ceramics [1,2,3,4,5,6,7]. Among them, ceramic has been widely used and actively investigated, especially with the development of computer-aided design/computer-aided manufacturing (CAD/CAM) technology. Thus far, dental ceramics that have been widely utilised in clinics include dental Empress and InCeram Alumina. However, these materials do not satisfy the physical properties, such as strength, required by dental clinics [1,2,3]. To improve the properties of these ceramics, research into zirconia is ongoing. Several studies on zirconia have found that this compound enhances material fracture strength and biocompatibility; it has been widely applied as a material for dental clinic-fabricated prostheses and artificial joints [8]. Zirconia ceramic prostheses have been increasingly used because patients demand better mechanical properties and aesthetics [9,10]. In addition, CAD/CAM technology has been applied to clinic care for dental restorations employing zirconia. However, the use of CAD/CAM to produce zirconia prostheses may produce rough scratches or form defects on the prosthesis surface, eventually leading to cracks and fracture. Furthermore, the fabrication of highly complex shapes using CAD/CAM is extremely difficult [10,11,12]. To overcome these disadvantages, research on additive manufacturing and formulations for additive manufacturing is actively being conducted. Additive manufacturing is a method that involves stacking the layers of materials using digital image processing techniques [13]. Recent research indicates that dental prostheses manufactured through additive manufacturing present acceptable density compared with those produced by milling or traditional methods [14,15,16,17,18].

Additive manufacturing includes spot scanning-based stereolithography, which uses a stereolithography apparatus (SLA), digital light processing (DLP), and Polyjet printing based on mask image projection [7]. Of these, SLA and DLP are technologies that print in layers using a photocuring reaction of photosensitive resins. The DLP technology stacks and hardens a formulated liquid photocurable resin through a photocuring process involving projected light according to the type and location of the shape to be moulded [19,20]. The main advantages of DLP are as follows: the printed product is highly dense and has a luminous surface, and the work speed is fast because the resin is moulded in face units. In addition, DLP has a faster production speed than the SLA process and enables the low-cost fabrication of samples with a small sample size [20,21]. For these reasons, the DLP system is considered suitable for additive manufacturing printers for dental ceramic production. However, despite the advances in this technology, preventing the formation of micro-cracks on the zirconia surface is difficult. The physical properties (e.g., strength) of the product produced by additive manufacturing are found to be extremely inadequate for actual clinical application [22]. To resolve this issue, a silane coupling agent can be added to the suspension for additive manufacturing. The agent combines with the surface of inorganic fillers or glass fibres that are mixed with the polymer on one side of the molecules, but has an affinity with the matrix resin on the other side. Dispersibility is enhanced, and the interfacial adhesiveness among inorganic fillers or between the glass fibre and matrix polymer improves. An organic coating based on a silane coupling agent is applied to enhance surface characteristics [23,24,25,26]. Its use was found to improve the strength of each sample. Studies indicate that the silane coupling agent improves the zirconia surface by promoting properties, such as surface strength and abrasion as well as friction resistance [27]. More importantly, silane coupling agents reduce the number of hydroxyl groups on the surface of the modified filler, increasing hydrophobicity and improving dispersion in polymer matrices and resins. This has been found to improve the strength of samples by limiting the hydrophilicity of the filler and increasing the reactivity of the filler or compatibility with the selected polymer depending on the type of silane functional group [28,29,30,31].

Methyltrimethoxysilane (MTMS), an organosilane with one methyl group and three hydrolyzable methoxy substituents, has been successfully used to impart hydrophobicity and oleophobicity to a variety of substrates. As previously reported, MTMS controls the hydrolysis conditions, so that the hydrolysis and coating processes are usually in simple ambient aqueous conditions, which represents an obvious advantage and is easily compatible with existing manufacturing processes [32].

In this study, the zirconia suspensions with a volume fraction of 52–56% without added silane were set as the control group [33], whereas those with the coupling agent were set as the experimental groups. The groups were prepared to compare the physical properties of zirconia specimens after sintering based on the incorporation or absence of a silane coupling agent. The null hypothesis of this study is that there is no difference in the physical and mechanical properties of the 3D printed zirconia samples with and without the addition of a silane coupling agent according to volume fraction.

## 2. Materials and Methods

### 2.1. Preparation of Zirconia Photocurable Suspension

**Figure 1:**
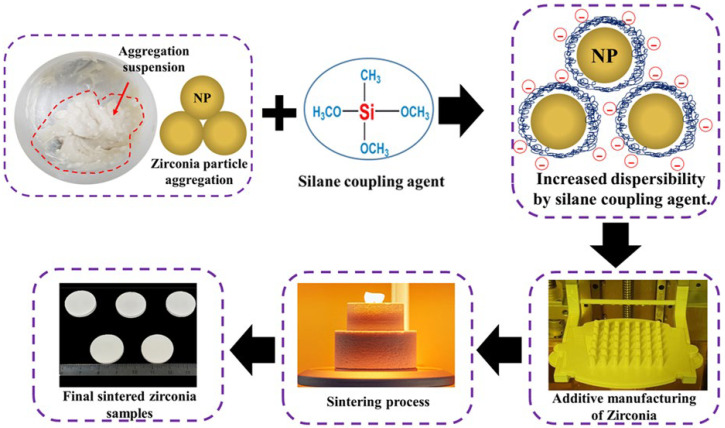
Schematic illustration of the experimental group process.

Commercially available zirconia powder (TZ-3Y, Tosho, Japan) with an average particle size of 310 nm was used. Three monomers (all obtained from Sigma Aldrich Inc., St. Louis, MO, USA), i.e., acrylic resin-based IBA (isobornyl acrylate), HDDA (1,6-hexanediol diacrylate), and PNPGDA (propoxylated neopentyl glycol diacrylate), were utilised to manufacture a photocurable binder. Other additives were a photoinitiator (Irgacure 819, Ciba Specialty Chemicals, Swiss), a dispersant to improve the dispersibility of zirconia powder binder (BYK-180, BYK Inc., Wesel, Germany), and a silane coupling agent (methyltrimethoxysilane coupling agent, MTMS, Duksan, Korea). MTMS mixed solution was prepared by stirring at a mass ratio of ethanol, distilled water, and MTMS (90:8:2) for 1 h. Then, 100 g of zirconia was mixed with the MTMS mixed solution. After adding the particles, it was ball milled for 24 h and then dried overnight at room temperature. The physical properties of the silane-modified zirconia particles were evaluated using FT-IR spectroscopy (Spectrum 400, Perkin Elmer, USA). The silane-modified zirconia powders were used to prepare suspensions of zirconia volume fractions of 52, 54, and 56 vol%. A planetary centrifugal mixer (ARV-310, Thinky Corp., Tokyo, Japan) was used to mix the suspension homogeneously. The groups were mainly divided into Z (control group) and ZS (experimental group with silane coupling agent); further, it was sub-divided into three control groups and three experimental groups based on zirconia volume fraction. The zirconia suspension compositions are summarised in Table 1. The detailed schematic illustration of the experimental group process was displayed in Figure 1.

### 2.2. Zirconia Samples Fabrication Using Additive Manufacturing

**Figure 2:**
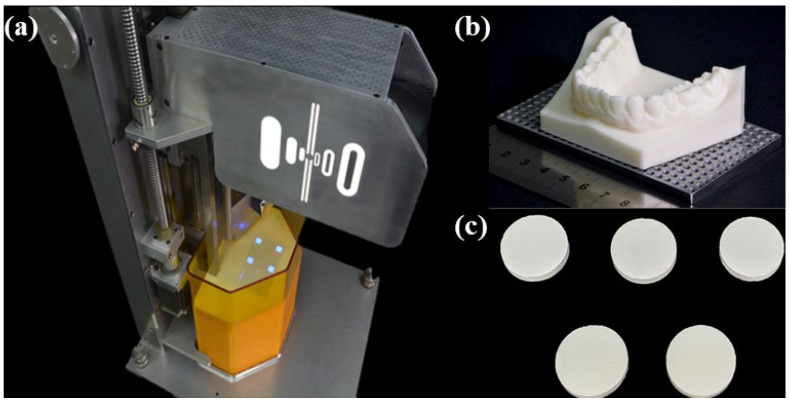
DLP machine used for preparing zirconia samples: (**a**) DLP machine curing formulations; (**b**) mandible and teeth model manufactured using DLP machine; (**c**) zirconia disk samples manufactured by DLP machine.

A DLP machine was used for light-curing the zirconia suspension (Octave Light R1, Octave Light Ltd., Shatin, Hongkong) (Figure 2); the waveband was 385 nm. Zirconia samples were additively manufactured by setting the thickness of each layer to 50 µm. The STL files of samples were designed using a three-dimensional (3D) modelling program (Solid Works 2016, Dassault Systemes SOLIDWORKS Corp., Waltham, MA, USA). Each sample was fabricated in the form of a disk with diameter and height of 20 and 2 mm, respectively.

### 2.3. Degreasing and Sintering of Zirconia Samples

**Figure 3:**
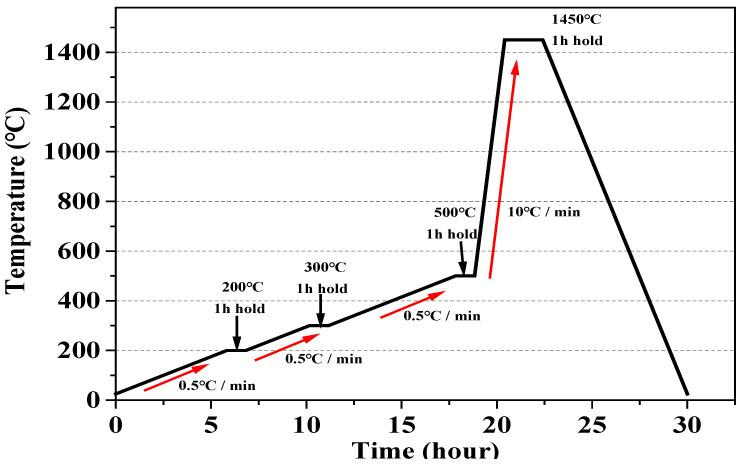
Sintering process of zirconia specimens (The red arrow indicates the heating rate.).

The additively manufactured zirconia samples were degreased and sintered using a sintering machine (DUOTRON PRO ex-6100, Zirconia Sintering Furnace, ADDIN Co., LTD, Suwon, Korea). The degreasing of each experimental group proceeded as follows. The temperature was gradually increased by 0.5 °C/min until it reached 200 °C and maintained for 1 h. Then, it was increased to 300 °C at 0.5 °C/min and again held for 1 h. Finally, the temperature was incrementally increased to 500 °C at 0.5 °C/min and maintained for 1 h. After the degreasing process, the sintering process proceeded as follows. The temperature was gradually increased to 1450 °C at 10 °C/min and held for 2 h. Then, the samples were naturally cooled (Figure 3) [26]. The final sintered zirconia samples were 14.5 and 2 mm in diameter and height, respectively.

### 2.4. Linear Shrinkage

The linear shrinkage of fabricated zirconia samples (*n* = 10) was measured using a vernier calliper (ASTM C 326: 2009). The diameter and height of samples were measured before and after sintering according to the volume fraction of zirconia. The following formula is used:

△X = {(X_1_ − X_2_)/X_1_} × 100, (1)

where X₁ and X₂ are the volume (mm^3^) of zirconia before and after sintering, respectively.

### 2.5. Relative Density Measurement

The relative density of zirconia samples was measured according to the volume fraction with reference to ISO 18754: 2007. After grinding the surface to reduce the influence of surface roughness, relative density is calculated using the following formula:

ρ = (m_1_/m_3_ − m_2_) × ρ_(Liq)_, (2)

where m_1_ is the dry mass (g), m_2_ is the underwater mass (g), m_3_ is water mass (g), and ρ_(Liq)_ is the density of liquid infiltration solution (density of water at room temperature = 1.0 g/m^3^); relative density (%) is calculated by (measured density/theoretical density) × 100.

### 2.6. Hardness

To measure the hardness of the final sintered zirconia samples (each with a diameter and height of 14.5 and 2 mm, respectively), these were fixed to multiple diamond plates and ground under constant pressure using grits in the order #400, #800, #1200, and #2400 (*n* = 10). The sample surface was finely ground using diamond pastes with 3 µm and 1 µm particles (MP-DS-50-0300, MTDI, Daejeon, Korea). The ground samples were pressed five times for 12 s with a force of 1 kgf (9.8 N) using a Micro-Vickers hardness tester (NOVA^®^ 130/240, INNOVATEST, Maastricht, Nederland) to determine the micro-hardness at each instance.

### 2.7. Biaxial Flexural Strength

The flexural strength of zirconia samples pertains to the bending stress that the samples can withstand when pressure is applied to them. The biaxial flexural strength of samples depending on the volume fraction was measured according to ISO 6872. Ten samples from each experimental group were measured by loading in a universal testing machine (RB Model 301 Unitech MTM R&B, Seongnam, Korea). The load was increased at a cross-head speed of 1 mm/min until the samples were completely fractured. Calculations are performed using the following formulae:

σ = − 0. 2387P (X − Y)/b2,(3)

X = (1 + v) ln(r2/r3)2 + [(1 − v)/2] (r2/r3) (2),(4)

Y = (1 + v) [1 + ln(r1/r3)2] + (1 − v) (r1/r3) (2), (5)

where σ is the biaxial flexural strength (MPa); P is the maximum load at fracture (Newtons); b is the sample thickness (mm); v is Poisson’s Ratio (v = 0.25); r1 is the radius of the circle supporting the load (mm); r2 is the radius of the circle to which the load is applied (mm); and r3 is the sample radius (mm).

### 2.8. Evaluation of Microstructure of Final Sintered Zirconia Samples

To observe the microstructure of zirconia samples, their surfaces were ground using diamond pastes with 3 µm and 1 µm particles (MP-DS-50-0300, MTDI, Daejeon, Korea). Then, they were cleaned with alcohol and distilled water in an ultrasonic cleaner (JAC-2010, KODO Technical Research Co., Hwaseong, Korea) for 20 min. To observe the cross-section, the samples were cleaned with alcohol and distilled water for 20 min. The platinum coating was applied to sample surfaces using a sputter coater (108 Auto, Cressington Scientific Instruments, Ltd., Oxhey, UK). After coating, the zirconia sample layers were observed using a scanning electron microscope (FE-SEM, JEOL, JSM-7500F, Tokyo, Japan).

### 2.9. Statistical Analysis

The sample size was evaluated using G-Power 3.1 software (University of Dusseldorf, Dusseldorf, Germany) [5]. The experimental results were statistically processed by one-way analysis of variance using the SPSS software package (SPSS Version 20.0, SPSS Inc., Chicago, IL, USA), and post tested with the Tukey HSD test. All results were tested for statistical significance with *p* < 0.05.

## 3. Results

### 3.1. FT-IR Analysis

**Figure 4:**
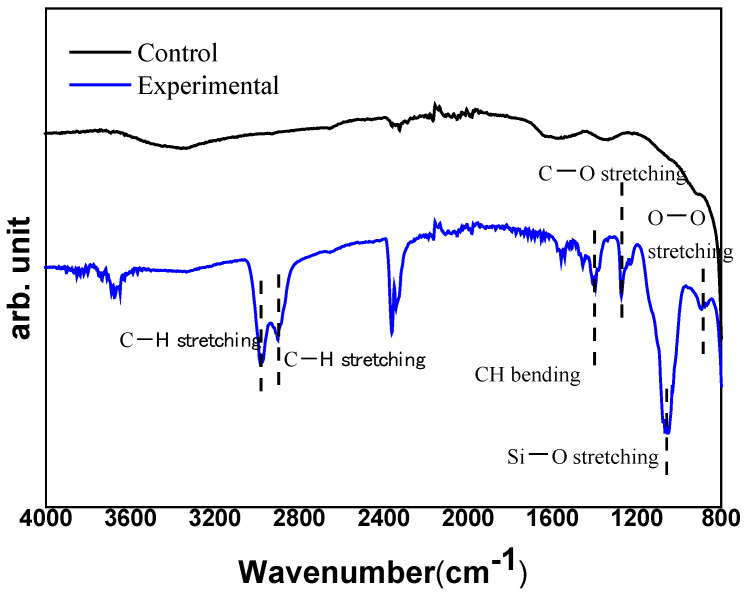
FT-IR spectra of the zirconia powder before and after silanization.

FT-IR spectroscopy was performed to determine the organic characterization, i.e., the effect of silanization of zirconia particles. As shown in Figure 4, the stretching vibration of -CH appears at 2983 cm^−1^ and 2900 cm^−1^, respectively. The bending vibration of –CH is 1405 cm^−1^. At 1270 cm^−1^ the stretching vibration of C–O appears, while the stretching vibration of –Si–O– attached to the –CH is at 1052 cm^−1^, the stretching vibration of –O–O– appears at 890 cm^−1^. From the results, it was confirmed that the zirconia particles were treated with the silane coupling agent, and a zirconia slurry was prepared using the treated powder.

### 3.2. Linear Shrinkage

**Figure 5:**
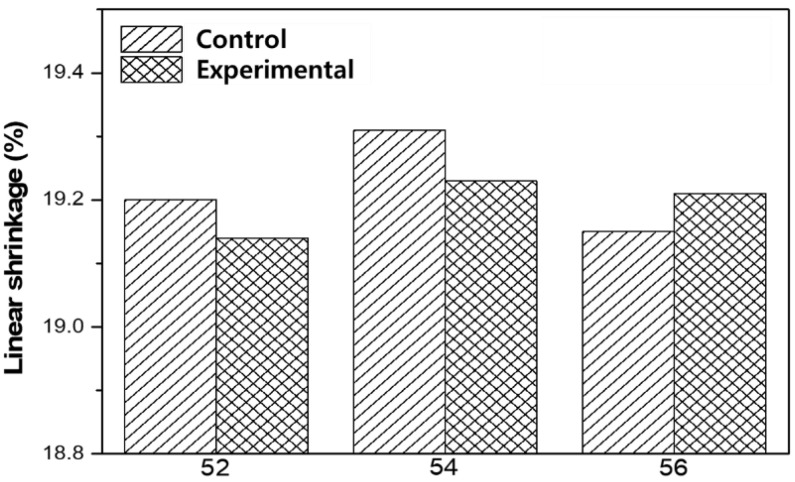
Comparison of linear shrinkage between control and experimental groups after final sintering.

The linear shrinkage of additively manufactured zirconia samples was measured according to the zirconia volume fraction by comparing them before and after sintering (Table 2 and Figure 5).

In the previous experiment, the shrinkage range of zirconia powder samples sintered at 1200–1400 °C was 19–20%; consequently, the average linear shrinkage of zirconia ceramic was found to be 18–25% [11]. The linear contraction rate of experimental and control groups exhibited a constant pattern of approximately 18–20%; however, no significant difference in the contraction rate between the two groups was observed. Moreover, it should be noted that in all groups, linear shrinkage was greater in height than in diameter. This is attributed to the occurrence of linear shrinkage in sample height during sintering, indicating that the action of gravitational force on height exceeds the effect of this force on diameter. Moreover, the greater linear shrinkage in height than in diameter was presumed to occur because the evaporation of resin in each layer at high temperatures caused the shrinkage of air gaps [34,35].

### 3.3. Relative Density

**Figure 6:**
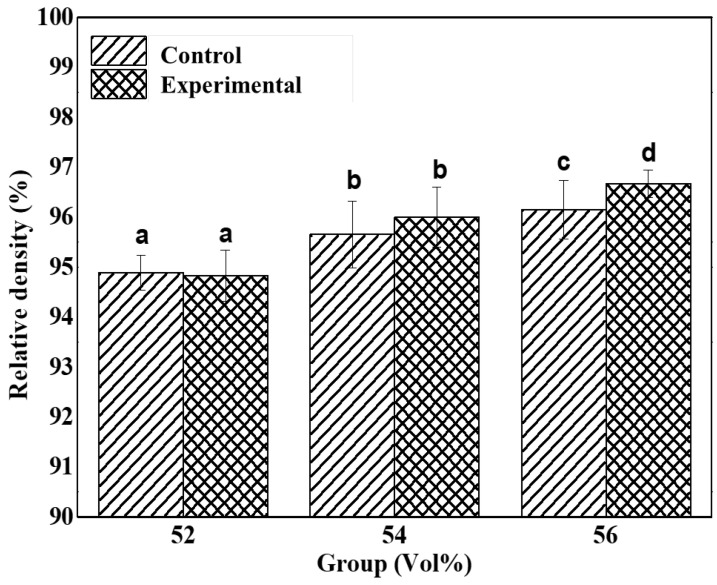
Comparison of relative density between control and experimental groups; different letters represent significant differences (*p* < 0.05).

The relative density values of additively manufactured zirconia samples in control groups and experimental groups are shown in Table 3 and Figure 6.

The difference between Z52, Z54, and Z56 was significant (*p* < 0.05). As for the experimental groups, the difference between ZS54 and ZS56 was insignificant, whereas that between these two and ZS52 was significant. Based on the addition of silane coupling agent, the difference between Z52 and ZS52 and that between Z54 and ZS54 did not significantly differ; however, a significant difference between Z56 and ZS56 (*p* > 0.05) was observed. Regardless of whether the silane coupling agent was added to the samples, the relative density increased as the volume fraction of zirconia increased. However, when a high-volume fraction of zirconia was added, density was further increased with the addition of a silane coupling agent. The silane coupling agent was presumed to enable the homogeneous dispersion of zirconia particles in the suspension. As a result, density was evenly distributed during particle growth [36]. The higher the relative density, the higher the sample density; hence, mechanical properties, such as strength, can be enhanced.

### 3.4. Hardness Measurement

**Figure 7:**
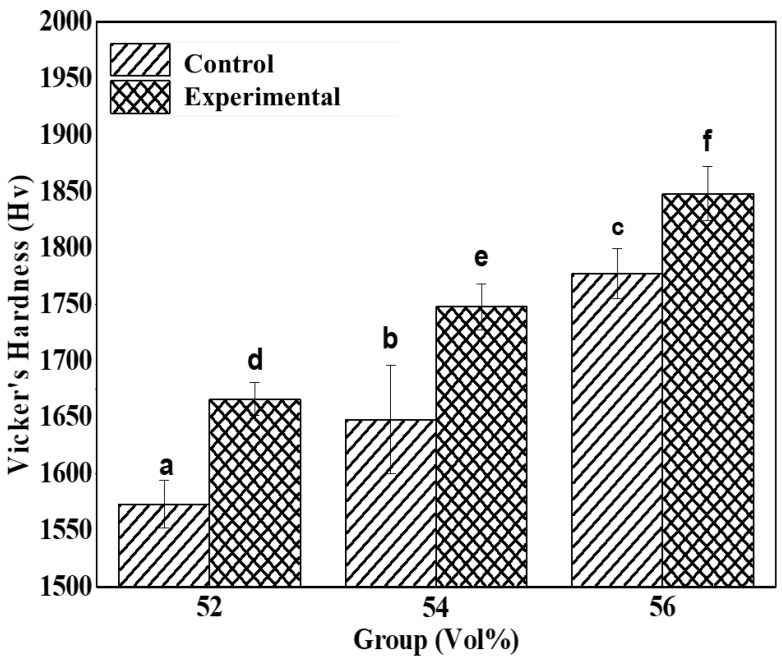
Comparison of Vickers hardness between control and experimental groups; different letters represent significant differences (*p* < 0.05).

The Vickers hardness evaluation results of control and experimental groups were shown in Table 4 and Figure 7.

All experimental groups significantly differed in average hardness (*p* < 0.05). All zirconia samples exhibited significant variations in Vickers average hardness (*p* < 0.05). Group ZS56, which had a zirconia volume fraction of 56 vol% and contained a silane coupling agent, exhibited the highest hardness value; it was followed by Z56, ZS54, ZS52, Z54, and Z52. The hardness values among these groups significantly differed (Figure 5). The addition of silane coupling agents to the samples promoted the homogeneous commixture of organic (photocurable polymers) and inorganic (zirconia) substances. As a result, dispersibility improved and hardness increased [21,28,36].

### 3.5. Biaxial Flexural Strength

**Figure 8:**
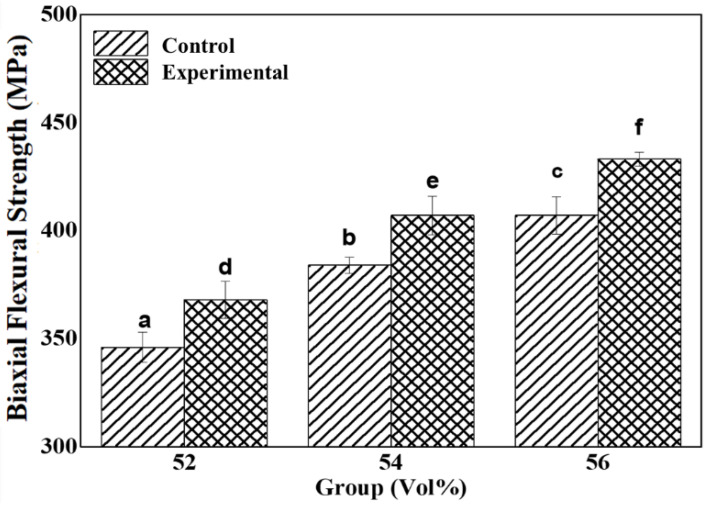
Comparison of biaxial flexural strength between control and experimental groups; different letters represent significant differences (*p* < 0.05).

From the biaxial flexural strength measurement of samples, the average biaxial flexural strength was displayed in Table 5 and Figure 8.

The biaxial flexural strengths between Z52 and ZS52, between Z54 and ZS54, and between Z56 and ZS56 significantly differed according to the added silane coupling agent (*p* <0.05). Similar to the results of Vickers hardness measurement, the following results are attributed to the commixture of photocuring polymer and zirconia promoted by the addition of a silane coupling agent, thereby increasing dispersibility [37]. Accordingly, the biaxial flexural strength of groups with the silane coupling agent increased.

### 3.6. Microstructure Evaluation

**Figure 9:**
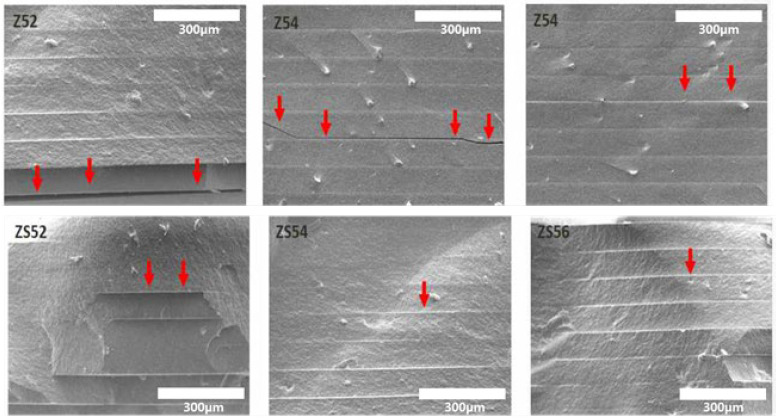
FE-SEM images of microstructure and layer-by-layer observation of the final sintered zirconia specimen (Red arrows indicate microcracks and pores).

The fractured sections of additively manufactured zirconia samples were observed using the scanning electron microscope for microstructure evaluation according to the zirconia volume fraction (Figure 9). The layers of additively manufactured zirconia samples were examined through this observation. The smaller the zirconia volume fraction, the more air gaps were observed (red arrows in Figure 9). The variations in relative density measurements were confirmed to be caused by these air gaps.

These observations are considered to support the measurements of low density and strength because of the occurrence of numerous air gaps in groups with no added silane coupling agent. In addition, the air gaps in groups with the added silane coupling agent were smaller in size compared with those in groups with no added coupling agent.

The images of the layers of additively manufactured zirconia samples were captured through scanning electron microscopy. The layers were observed to be smaller and more constant in groups with the added silane coupling agent compared with those without the agent. Moreover, the proportion of air gaps caused by the debinding of zirconia samples during sintering was found to decrease and then remain constant. If the air gaps were not constant, micro-cracks were more likely to occur due to external forces that may degrade strength, hardness, and physical properties [33,38]. If zirconia-based prostheses are applied in actual dental clinical practice, the foregoing can increase the possibility of prosthesis breakage caused by the masticatory force of patients.

Therefore, considering that the air gaps are reduced and remain constant, improved strength can be expected from additively manufactured zirconia with the added silane coupling agent.

This study demonstrated the effect of silane on the volume fraction of zirconia. However, future experiments are needed to evaluate the dispersion of the silane-modified zirconia particles in the suspension by varying experimental parameters associated with the salinisation of zirconia particles, such as pH, solution composition, and hydrolysis time.

## 4. Conclusions

In this study, the preparation of zirconia suspensions with the addition of a silane coupling agent (approximately 2% of zirconia volume fraction) for DLP additive manufacturing technology has been demonstrated. It was established that the density of the printed zirconia samples significantly increased with an increase in the zirconia volume fraction (52, 54, and 56 vol%). Interestingly, the experimental group (ZS56) with the addition of silane coupling agent to the suspension of 56 vol% exhibits improved strength and hardness (5–6%) compared to those without silane coupling agent (control group). However, it is essential to focus future research activities on enhancing the dispersibility of zirconia suspension using the silane coupling agent for DLP printing. In future experiments, we plan to evaluate the dispersion stability of the zirconia suspension by varying conditions, such as pH control, solution composition, and hydrolysis time, for the encapsulation of the zirconia particles with the silane coupling agent.

## Figures and Tables

**Table 1:** Experimental groups in this study.

Group		Zirconia Powder	Acrylic Monomers			Additive Agents		Total wt (vol)%
			IBA	HDDA	PNPGDA	Dispersant	Silane Coupling Agent	
Control	Z52	86.45 (52.00)	9.23 (33.43)			4.32(14.57)	0	100
	Z54	87.34 (54.00)	8.29 (30.87)			4.37(15.13)	0	100
	Z56	88.19 (56.00)	7.41 (28.32)			4.41(15.68)	0	100
Experimental	ZS52	86.45 (52.00)	8.64 (30.30)			3.27(11.65)	1.64 (6.05)	100
	ZS54	87.34 (54.00)	7.68 (27.62)			3.32(12.10)	1.66 (6.28)	100
	ZS56	88.19 (56.00)	6.74 (24.94)			3.38(12.54)	1.69 (6.52)	100

**Table 2:** Average linear shrinkage of printed zirconia sample.

Groups	Linear Shrinkage (%)	
	Control	Experimental
Z52	19.20 ± 0.31	19.14 ± 0.17
Z54	19.31 ± 0.23	19.23 ± 0.20
Z56	19.15 ± 0.29	19.21 ± 0.09

**Table 3:** Relative density of printed zirconia; different letters represent significant differences (*p* < 0.05).

Groups	Relative Density (%)	
	Control	Experimental
Z52	94.89 ± 0.35 ^a^	19.14 ± 0.09 ^a^
Z54	95.65 ± 0.67 ^b^	19.06 ± 0.10 ^b^
Z56	96.15 ± 0.59 ^c^	19.05 ± 0.03 ^d^

**Table 4:** Vickers hardness of printed zirconia; different letters represent significant differences (*p* < 0.05).

Groups	Vickers hardness (HV)	
	Control	Experimental
Z52	1573 ± 21.31 ^a^	1666 ± 14.61 ^d^
Z54	1648 ± 48.17 ^b^	1748 ± 20.33 ^e^
Z56	1777 ± 22.19 ^c^	1848 ± 24.04 ^f^

**Table 5:** Vickers hardness of printed zirconia; different letters represent significant differences (*p* < 0.05).

Group	Flexural Strength (MPa)	
	Control	Experimental
Z52	346 ± 6.96 ^a^	368 ± 8.62 ^d^
Z54	384 ± 3.68 ^b^	407 ± 8.87 ^e^
Z56	407 ± 8.73 ^c^	433 ± 3.27 ^f^

## Data Availability

All the data have been illustrated in the manuscript.
